# The Impact of Rapid Weight Regain on Fight Outcomes in Bellator Mixed Martial Arts Athletes

**DOI:** 10.7759/cureus.77785

**Published:** 2025-01-21

**Authors:** Corey A Peacock, Peter Byers, Tobin Silver, Joey Antonio, Gabriel J Sanders, Antonella Schwarz, Lauren Stern

**Affiliations:** 1 Exercise and Sport Science, Nova Southeastern University, Davie, USA; 2 Exercise Science, Nova Southeastern University, Davie, USA; 3 Exercise Science, University of Cincinnati, Cincinnati, USA; 4 Exercise Science, Barry University, Miami, USA; 5 Dr. Kiran C. Patel College of Osteopathic Medicine, Nova Southeastern University Dr. Kiran C. Patel College of Osteopathic Medicine, Davie, USA

**Keywords:** competition, fighting, mma, weight, winning

## Abstract

Objectives: This study aimed to evaluate the effect of rapid weight regain on the fight outcomes in professional mixed martial arts (MMA) athletes competing in Bellator.

Materials and methods: Twenty fighters (16 male and four female fighters) were included in the analysis. Official weigh-in and fight-night weights were recorded, and percentage weight regain was calculated. Fighters were divided into groups based on their percentage weight regain (<10% vs. ≥10%). Descriptive statistics were calculated, and independent t-tests and logistic regression were employed.

Results: The results indicated that while fighters significantly regained weight between weigh-ins and fight-night, this weight gain did not significantly (p ≥ 0.05) predict fight outcomes.

Conclusion: These findings suggest that weight regain may not provide a competitive advantage in Bellator MMA athletes.

## Introduction

In combat sports like mixed martial arts (MMA), athletes often engage in rapid weight loss (RWL) and rapid weight regain (RWRG) strategies to gain a physical advantage during competition. By employing weight-cutting strategies, athletes can ideally compete in a lower weight class while regaining lost weight before their bout [1,2]. This strategy is prevalent and has been documented in professional MMA athletes including those competing for the Ultimate Fighting Championship (UFC), Bellator MMA, and many other professional MMA athletes [3-5]. The theory is that regaining weight provides a physical advantage, but current literature on the relationship between RWRG and fight outcomes is inconsistent. Some studies suggest that greater weight regain relates to better performance, while others state no significant impact [3,6-8]. Additionally, sex differences in RWRG have demonstrated that male fighters often regain more weight than female fighters on fight-night [9,10]. While RWL and RWRG practices have become recognized among MMA athletes, the impact of weight regain on fight outcomes remains unclear. It was found that RWRG in UFC athletes did not impact fight outcomes, but this has yet to be explored in Bellator MMA athletes [11]. This study plans to investigate whether the percentage of RWRG predicts fight outcomes in Bellator MMA athletes. Bellator MMA is a professional MMA organization that organizes events worldwide where athletes compete across various weight classes. Bellator MMA is considered a major organization commissioned by governing bodies including the California State Athletic Commission (CSAC). Based on previous findings, we hypothesize that weight regain will not significantly impact fight outcomes.

## Materials and methods

Twenty professional Bellator MMA athletes who competed in a sanctioned event in California, USA, were included in this study. All athletes had their body mass measured using the official CSAC calibrated scale. CSAC officials oversaw both the official and fight-night weigh-ins. The official weigh-in took place between 9:00 am and 11:00 am, approximately 36 hours before the competition. Upon arrival at the arena for the competition, athletes were weighed to record their fight-night weight. The fighters were included in the study as participation was mandatory based on CSAC event rules. The data, which were publicly available as part of official event records, were deidentified before analysis. The study protocol was reviewed and deemed in compliance by the institutional review board (IRB Protocol: 2024-142), and informed consent was not required due to the deidentified nature of the dataset. Descriptive statistics were calculated for age, height, weigh-in weight, fight-night weight, and percentage weight regain. Independent t-tests compared these variables between the male and female fighters. A logistic regression model assessed whether percentage weight regains predicted fight outcomes (win or loss). Fighters were categorized into two groups: those who regained <10% of their weight and those who regained ≥10%; 10% represents the average weight regain observed in professional fighters and was previously employed in prior research examining UFC fighters and fight outcomes [11]. It is worth noting that the dataset included an equal distribution of men and women in each group (<10% males = 8, females = 2; ≥10% males = 8, females = 2). All analyses were conducted using SPSS version 29 (IBM Corp., Armonk, NY), with a significance set at p ≤ 0.05.

## Results

**Figure 1:**
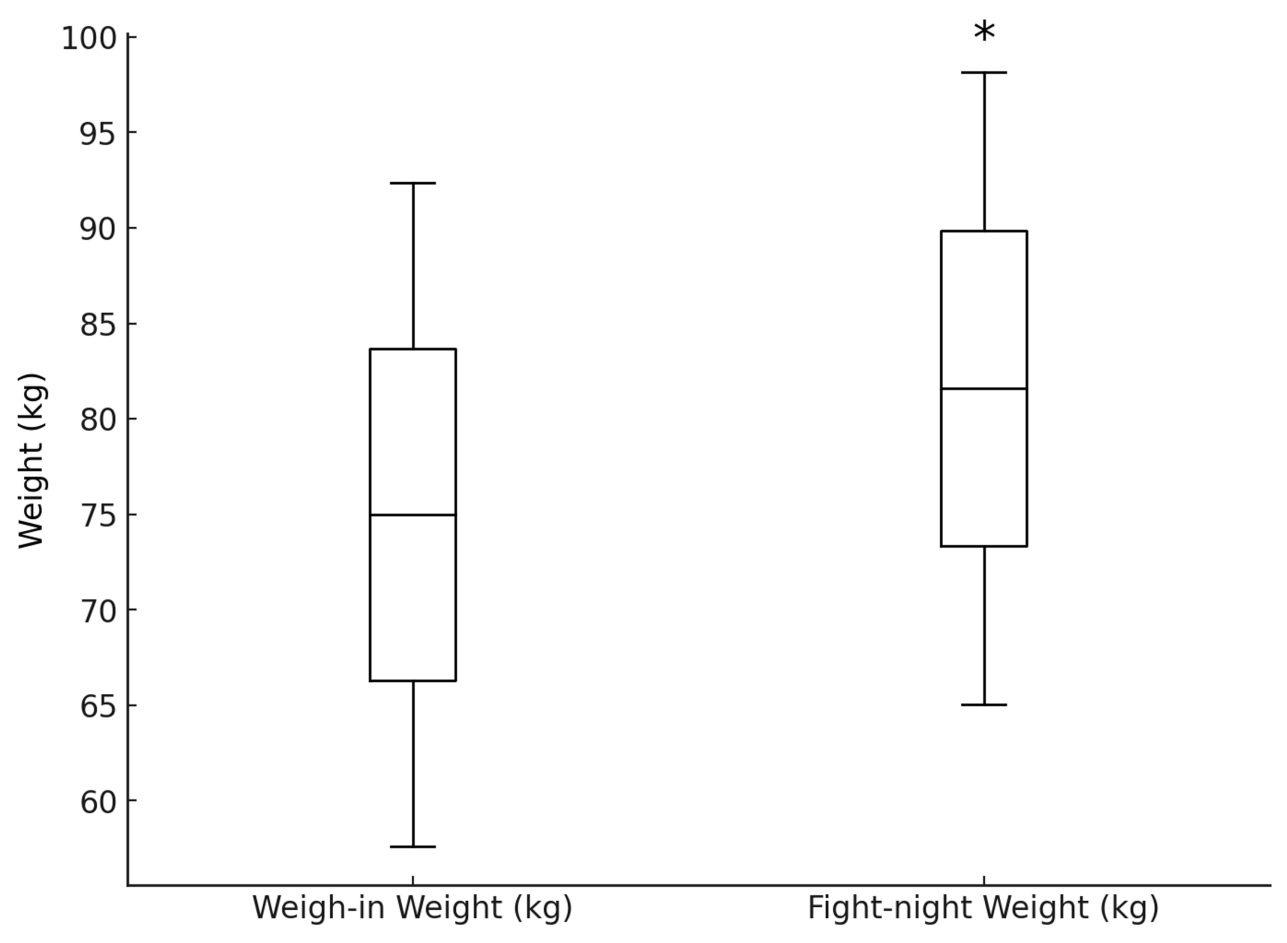
Comparison of weigh-in and fight-night weights ^*^Significance set at p ≤ 0.05

The 20 fighters (30.00 ± 3.81 years, 177.20 ± 9.42 cm) demonstrated an average percentage weight regain of 9.47 ± 4.57%. A significant difference in weight was observed between the weigh-in and fight-night weights (p < 0.001) (Figure 1). Significant differences were found between the male and female fighters in height (p = 0.006, d = 2.45, power = 0.99), weigh-in weight (p < 0.001, d = 2.26, power = 0.97), and fight-night weight (p < 0.001, d = 2.60, power = 0.99). It is worth noting that weight class distribution was greater in male fighters when compared with female fighters resulting in the difference in weigh-in and fight-night weights. The percentage weight gain was nonsignificant and has a very low power suggesting insufficient sample size for this particular variable (p = 0.522, d = -0.42, power = 0.11) (Table 1).

**Table 1:** Descriptive statistics by male and female ^*^Significance set at p ≤ 0.05

Variable	Male (n = 16)	Female (n = 4)	p value
Age (years)	29.75 ± 3.84	31.50 ± 4.51	0.471
Height (cm)	180.19 ± 6.94	164.00 ± 3.56	0.006^*^
Weigh-in weight (kg)	80.00 ± 13.50	57.91 ± 2.23	<0.001^*^
Fight-night weight (kg)	87.23 ± 12.92	62.30 ± 1.60	<0.001^*^
Percentage weight gain (%)	9.80 ± 4.83	7.81 ± 1.23	0.522

Logistic regression analysis revealed that percentage weight regain was not a significant predictor of fight outcomes (win and loss) (p = 0.798). Fighters who regained ≥10% of their weight were not significantly more likely to win than those who regained <10%; however, methods of victory were not analyzed. The distribution of wins and losses between these two groups was nearly even, further supporting the lack of association between the RWRG and fight success (Table 2).

**Table 2:** Percent change and performance outcome

Fighter	% Change	Result	Sex
1	12.1	Win	Male
2	12.4	Loss	Male
3	10.8	Win	Male
4	10.7	Loss	Male
5	9.3	Win	Male
6	2.9	Loss	Male
7	8.3	Win	Female
8	5.3	Loss	Female
9	8.9	Win	Male
10	14	Loss	Male
11	3.7	Win	Male
12	12.5	Loss	Male
13	7.6	Win	Male
14	15	Loss	Male
15	12.7	Win	Male
16	8.6	Loss	Male
17	0.6	Win	Male
18	2.9	Loss	Male
19	18.1	Win	Female
20	12.9	Loss	Female

## Discussion

This study explored the relationship between the RWRG and fight outcomes in Bellator MMA athletes. Although fighters significantly regained weight between weigh-ins and fight-night, the amount of weight regained did not significantly predict whether a fighter would win or lose. These findings align with previous studies including UFC athletes, which also found no correlation between RWRG and competitive success [3,6,11,12]. On average, Bellator athletes regained 9.47% of their body weight, a practice consistent with previous research in combat sports [3,4]. However, the results support the hypothesis that regaining more weight would not lead to a competitive advantage. Fighters who regained ≥10% of their body weight after the weigh-in were no more likely to win than those who regained less. This is consistent with previous literature, which also showed that RWRG did not appear to impact outcomes in UFC athletes [11]. Our findings align with prior research indicating that while RWRG is common, it may not be the critical factor influencing the outcome of a fight [8,13,14]. A limitation of this study is the small sample size, particularly for female athletes, which restricts the generalizability of the findings. It is also worth noting that female athletes only represented two weight classes and male athletes represented five weight classes, which may have impacted findings such as weigh-in differences. Current research by the investigators is aimed at including larger samples across various MMA organizations to better explore the effects of weight regain on performance. Furthermore, additional research aims to analyze methods of victory such as knockouts, technical knockouts, and decisions by points. This may prove more impactful than simply wins and loses. Finally, this study did not collect data on weigh-in strategies or the specific methods by which weight was regained after weigh-ins. These factors may have played a significant role in influencing match outcomes and represent an additional limitation that future research should address. In conclusion, while RWRG is common among Bellator MMA athletes, it does not significantly impact fight outcomes for this particular dataset.

## Conclusions

In conclusion, this study highlights that while RWRG is a prevalent practice among Bellator MMA athletes, it does not statistically influence fight outcomes. These findings are similar to previous research on similar populations, suggesting that the competitive advantage often associated to RWRG may not be prevalent. Despite the significant weight changes observed between weigh-ins and fight-night, these shifts were not predictive of success during competition. The study underscores the need for further investigation, particularly between organizations to comprehensively understand the role of RWRG in MMA. These insights may inform future discussions on weight management practices and athlete safety by sport performance professionals.
